# Zn- and Co-based layered double hydroxides: prediction of the *a* parameter from the fraction of trivalent cations and *vice versa*

**DOI:** 10.1107/S2052519213017545

**Published:** 2013-07-18

**Authors:** Ian G. Richardson

**Affiliations:** a School of Civil Engineering, University of Leeds, LS2 9JT Leeds, England

**Keywords:** layered double hydroxide

## Abstract

A recently proposed method to calculate the *a* parameter of the unit cell of layered double hydroxides from the fraction of trivalent cations is extended to Zn- and Co-based phases. It is shown to be useful as a sanity test for extant and future structure determinations and computer-simulation studies.

Layered double hydroxide (LDH) phases are derived from layered single hydroxides [*i.e.* β-*M*(OH)_2_ phases] by the substitution of a fraction (*x*) of the divalent cations by trivalent cations. Synthetic preparations are studied widely because of their use in a wide range of applications (Cavani *et al.*, 1991 ▶) and there are many natural LDH phases (Mills *et al.*, 2012 ▶). The author has recently derived equations that enable calculation of *x* from the *a* parameter of the unit cell of LDH phases and *vice versa*, whichever is known with most confidence (Richardson, 2013 ▶). The version for phases that have just one type of trivalent cation is given in equation (1).
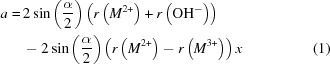
In equation (1): α is the angle between two oxygen sites and the metal site in an *M*—O octahedron of the main layer of the LDH phase when the two O atoms are in the same basal plane; *r*(*M^n^* ^+^) is the effective ionic radius of a cation that is in sixfold coordination; *r*(OH^–^) is the effective radius of the O atom of the hydroxyl ions that are at the vertices of the *M*—O octahedron. The values used for *r*(*M^n^* ^+^) are those given in the column labelled ‘IR’ in Table 1 of Shannon (1976 ▶) and *r*(OH^−^) was shown to have a value of 1.365 Å (Richardson, 2013 ▶).

The first part of the right-hand side of equation (1) was shown to be equal to the *a* parameter of an *M*(OH)_2_-type phase, 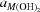, and so the *a* parameter for the layered double hydroxide (LDH), 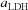, can be expressed as in equation (2), which is essentially a statement of Vegard’s Law, *i.e.* that in a solid solution series there is a linear relation between the lattice constant and composition (Vegard, 1921 ▶; West, 1984 ▶; Denton & Ashcroft, 1991 ▶).
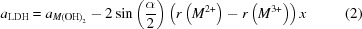
The *M*(OH)_2_-type phase was shown to be an α form of divalent metal hydroxide (Feitknecht, 1938 ▶) rather than the β-*M*(OH)_2_ polymorph [it is not a *M*(OH)_2_ polymorph because it has the composition *M*(OH)_2_·*m*H_2_O]. Values for the bond angle α were determined for Ni- and Mg-based LDH phases by fitting equation (1) to extensive sets of *a*–*x* data compiled from the literature, which produced values of 97.83 and 97.41° for Ni- and Mg-based phases respectively. These values were shown to be independent of the type of trivalent cation and of the type of interlayer anion; *i.e.* the extent to which the metal–oxygen octahedra are squashed is determined solely by the type of divalent cation. The determination of the values for α means that equation (1) can be used as a sanity test for the results of extant and future computer-simulation studies and crystal structure determinations for Ni- and Mg-based preparations where *x* has any value (including *x* = 0) and that have seemingly any type of trivalent cation.

The same process as detailed in Richardson (2013 ▶) for Ni- and Mg-based LDH systems can be applied to others based on different divalent cations, although the successful determination of a value for α of course depends on the quantity and quality of the data that are available in the literature. Plots of the *a* parameter against *x* for Zn–Al and Co–Al LDH preparations, both with a number of different anions in the interlayer, are shown in Figs. 1 ▶ and 2 ▶, respectively. The type of interlayer anion does not appear to affect the value of the *a* parameter, which was also the case for the Mg- and Ni-based preparations considered in Richardson (2013 ▶). These data sets both highlight one of the reasons why the procedure outlined in Richardson (2013 ▶) is useful. The arrows on the figures indicate groups of data points that correspond to values of *x* equal to 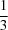 or 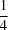. These two compositions are very commonly studied because they are the largest values of *x* that correspond to hexagonal ordered distributions of trivalent cations in the octahedral layer (Brindley & Kikkawa, 1979 ▶). The fact that data points occur at these values in vertical stacks on the figures suggests strongly that the compositions for many of those points are not correct, either because the compositions of the initial mix used in the synthesis procedure were simply assumed to apply to the solids and were not checked, or because of incorrect interpretation of bulk analyses where multi-phase mixtures were present. Since second phases can be amorphous or crystalline it may be necessary to measure the actual composition of the LDH crystals, for example by microanalysis in a transmission electron microscope. Errors are also possible with the measurement of the *a* parameter, particularly where the (110) peak on the X-ray diffraction pattern is broadened due to a small average crystal size [*a* is calculated from the *d* spacing of the (110) peak, *a* = 2 × *d*_110_], which is of course why a proven link between *a* and *x* provides a useful check. The two unfilled square symbols on Fig. 1 ▶ represent points for the Zn–Al–CO_3_ phase zaccagnaite from the crystal-structure determinations of Merlino & Orlandi (2001 ▶) and Lozano *et al.* (2012 ▶). The positions of both points suggest that the compositions are possibly in error, although they were both determined by electron microprobe analysis. It is perhaps possible that in both cases there was some aluminium hydroxide present within the X-ray generation volume, which would result in an increased value of *x*. This is particularly plausible in the case of Lozano *et al.* because those authors did discuss the presence of nordstrandite in their sample.

As in Figs. 4(*a*) and 6(*a*) of Richardson (2013 ▶) the full lines on Figs. 1 ▶ and 2 ▶ are linear regression fits, but in these cases the range for the fitting had to be restricted to avoid some of the stacked data points discussed above. Despite the obvious limitations of the data, it is nevertheless clear that the general trends are consistent with equation (2) and that the values of *a* at *x* = 0 again do not correspond to the β polymorph of the respective *M*(OH)_2_ phase, which are represented on the figures by the filled diamonds. Substitution of the value established in Richardson (2013 ▶) for *r*(OH^−^) (*i.e.* 1.365 Å) together with the effective ionic radius for the *M* ^2+^ ions in sixfold coordination (from Shannon, 1976 ▶) into equation (1) allows values for α to be derived by adjusting it to give the best match with the linear regression lines for the Zn–Al and Co–Al LDH data in Figs. 1 ▶ and 2 ▶. The results are shown by the dashed lines on those figures that fall close to the regression lines (which are the full lines); the values of α obtained in this way are 97.18 and 97.12° for the Zn–Al and Co–Al systems, respectively. The open diamonds can again be taken to represent a theoretical α form of the divalent metal hydroxide, *M*(OH)_2_·H_2_O, and so the *a* parameters for these α-*M*(OH)_2_ phases are obtained by substituting *x* = 0, *r*(OH^−^) = 1.365 Å and α = 97.18 or 97.12° into equation (1), which gives 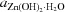 = 3.157 Å, and 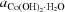 = 3.164 Å. The values of α determined here for Zn- and Co-based LDH phases could be used to produce model crystal structures, as done in Richardson (2013 ▶) for Ni- and Mg-based systems, but unfortunately there are currently insufficiently precise data in the literature to allow prediction of the *c* parameter.

Interestingly, the values of α determined for the four divalent metal systems considered here and in Richardson (2013 ▶) produce a very good linear relationship with *r*(*M* ^2+^), which at first sight would seem to offer the prospect of extending the procedure to other systems for which there are limited data; the equation is α = 106.73 − [12.913 × *r*(*M* ^2+^)], which is shown as a dotted line in Fig. 3 ▶ (*r* ^2^ = 0.99). However, the data for the single hydroxides do not show the same sort of relationship (Fig. 3 ▶) and so the strong correlation for the LDH systems is perhaps fortuitous. The value of α determined for Mg-based LDH phases is quite close to the value for β-Mg(OH)_2_ (*i.e.* brucite), but the respective values for the Ni-, Zn- and Co-based phases are not particularly close, although there is a good linear relationship for those three systems. This means that at the moment it is not possible to derive a value for α for an LDH phase from the value for the corresponding single hydroxide. However, since the positions of the calculated lines on *a*–*x* plots (*e.g.* Figs. 1 ▶ and 2 ▶) are very sensitive to small changes in both α and *r*(OH^–^), it is possible that future improvements in the quality and precision of the various data might result in a satisfactory crystal-chemical explanation for any similarities or differences between the values for the single and double hydroxides (*i.e.* in addition to the difference in the value of the effective radius of the O atom of the hydroxyl ion, as established in Richardson, 2013 ▶).

In principle, for LDH systems for which there are few data or data of uncertain quality, EXAFS (extended X-ray absorption fine structure) could be used as a method to determine a value for α that avoids the need to synthesize a series of phases with variable *x*: *d*(*M*—O) and *d*(*M*—*M*) (which is equal to the *a* parameter) can be determined using EXAFS, which can then be used in equation (13) of Richardson (2013 ▶) to calculate a value for α. As examples, data for Zn-based systems can be found in Leroux, Adachi-Pagano *et al.* (2001 ▶), Funke *et al.* (2005 ▶) and Aimoz *et al.* (2012 ▶); for Co-based systems in O’Day *et al.* (1994 ▶), Leroux, Moujahid *et al.* (2001 ▶) and Intissar *et al.* (2004 ▶); for Ni-based systems in Pandya *et al.* (1990 ▶), d’Espinose de la Caillerie *et al.* (1995 ▶), Scheidegger *et al.* (1998 ▶) and Vespa *et al.* (2006 ▶); and data for Mg-based systems in Bellotto *et al.* (1996 ▶). The data in these articles all result in values of α > 90°, which indicates that the octahedra are squashed, but unfortunately the precision of the data as reported is not sufficiently high to be useful in calculations of the *a* parameter.

In summary, the treatment given in Richardson (2013 ▶) that enables calculation of the *a* parameter of LDH phases from the composition (*x*) has been extended successfully to Zn- and Co-based phases, which should be useful as a sanity test for extant and future structure determinations and computer simulation studies that are concerned with those systems. The values of α for the four divalent metal systems considered here and in Richardson (2013 ▶) are 97.83, 97.41, 97.18 and 97.12° for Ni-, Mg-, Zn- and Co-based phases, respectively. These are the values that should be used in equation (1). Since α is between 97 and 98° for all four systems it is clear why the literature treatment of the crystal chemistry of LDH phases had been unsatisfactory until the author’s recent paper because the equations that have been reported previously have always been for regular octahedra (*i.e.* α = 90°).

## Figures and Tables

**Figure 1:**
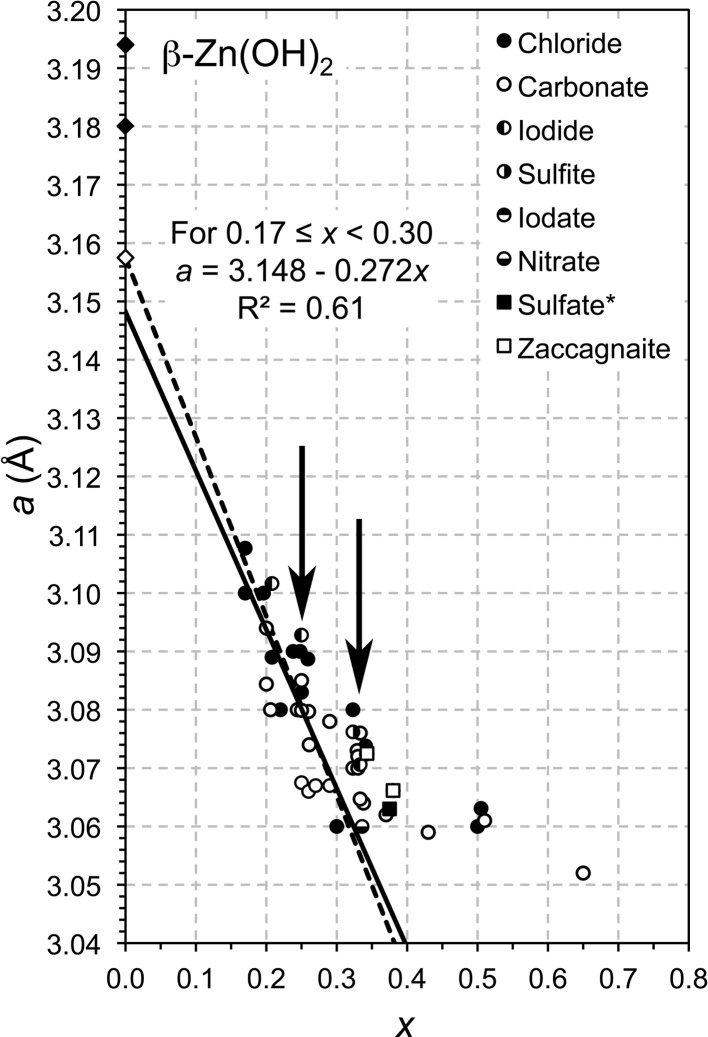
Plot of the *a* parameter against *x* for a range of Zn—Al LDH phases reported in the literature. The data involve a variety of interlayer anions, which are represented by different symbols (that are identified in the figure legend). The full line is the result of the linear regression analysis of the data. The filled diamonds represent the β polymorph of Zn(OH)_2_; the upper filled diamond is from Baneyeva and Popova’s structure (Baneyeva & Popova, 1969 ▶) and the lower one is from Richardson’s (2013 ▶) structure. The dashed line represents the values of *a* calculated from theory [using equation (1)]; the open diamond can be taken to represent a theoretical α form of Zn hydroxide [Zn(OH)_2_·H_2_O]. The data are from: Aimoz *et al.* (2012 ▶), Barriga *et al.* (1998 ▶), Benito *et al.* (2008 ▶), Rojas Delgado *et al.* (2008 ▶), Johnson & Glasser (2003 ▶), Kooli *et al.* (1997 ▶), Lakraimi *et al.* (2006 ▶), Leroux, Adachi-Pagano *et al.* (2001 ▶), Lozano *et al.* (2012 ▶) (zaccagnaite), Merlino & Orlandi (2001 ▶) (zaccagnaite), Miyata (1975 ▶), Radha *et al.* (2007*a* ▶), Radha *et al.* (2011 ▶), Seftel *et al.* (2008 ▶) (the *a* parameter for these data was calculated from the XRD patterns because the values given in their Table 1 are not consistent with the patterns); Thevenot *et al.* (1989 ▶), Troutier-Thuilliez *et al.* (2009 ▶), Vieira *et al.* (2009 ▶), Witzke & Raade (2000 ▶) (*zincwoodwardite).

**Figure 2:**
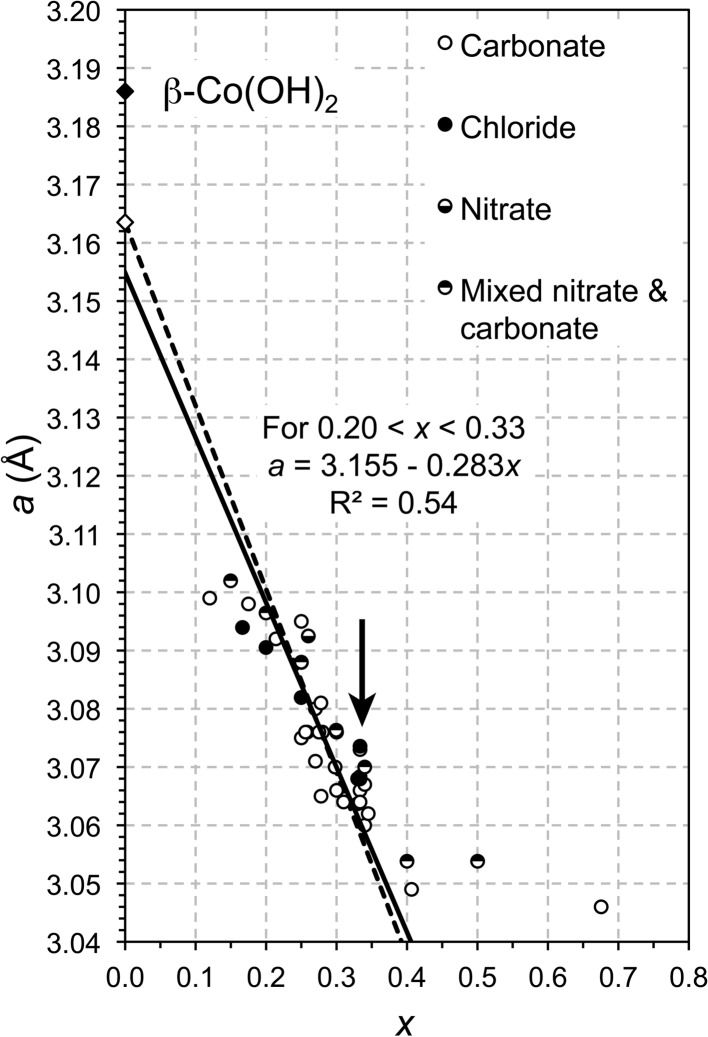
Plot of the *a* parameter against *x* for a range of Co–Al LDH phases reported in the literature. The data involve a variety of interlayer anions, which are represented by different symbols (that are identified in the figure legend). The full line is the result of the linear regression analysis of the data. The filled diamond represents the β polymorph of Co(OH)_2_ (Pertlik, 1999 ▶). The dashed line represents the values of *a* calculated from theory [using equation (1)]; the open diamond can be taken to represent a theoretical α form of Co hydroxide [Co(OH)_2_·H_2_O]. The data are from: Gabrovska *et al.* (2011 ▶), Géraud *et al.* (2008 ▶), Herrero *et al.* (2007 ▶), Intissar *et al.* (2003 ▶), Johnsen *et al.* (2010 ▶), Johnsen & Norby (2008 ▶), Johnson & Glasser (2003 ▶), Kannan *et al.* (1995 ▶), Kannan & Swamy (1999 ▶), Leroux, Moujahid *et al.* (2001 ▶), Liu *et al.* (2006 ▶), Luo & Dahn (2009 ▶), Pérez-Ramírez *et al.* (2001 ▶), Radha *et al.* (2007*b* ▶), Ribet *et al.* (1999 ▶), Rives *et al.* (2003 ▶), Sato *et al.* (1988 ▶), Vieira *et al.* (2009 ▶).

**Figure 3:**
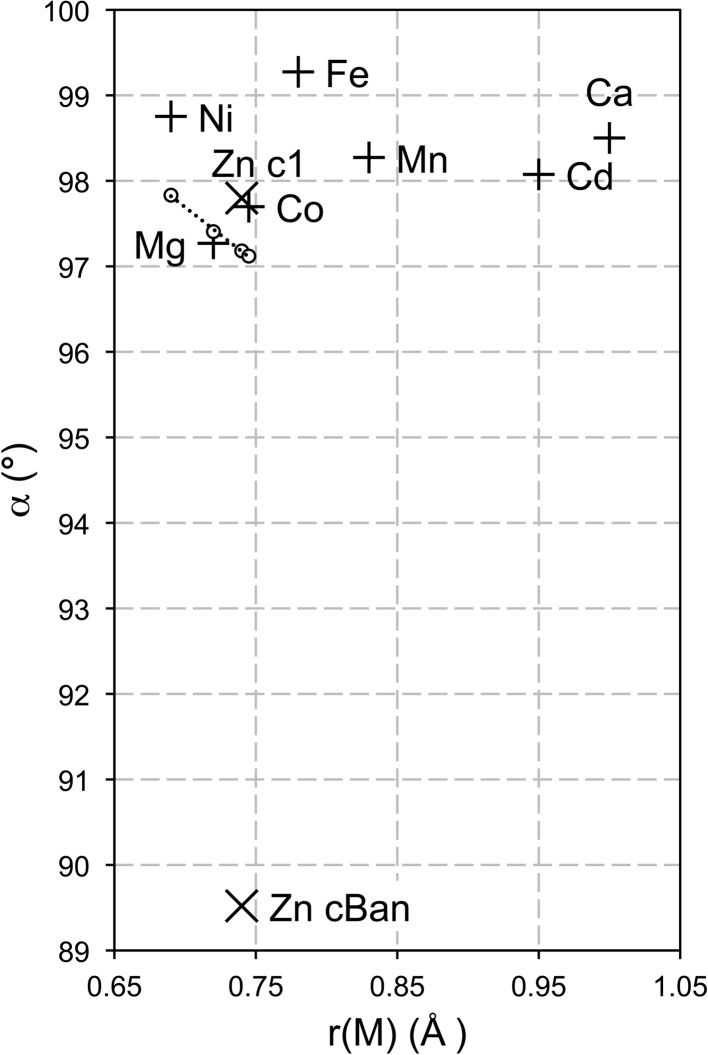
Plot of the bond angle α against *r*(*M*) for β-*M*(OH)_2_ phases and for Ni-, Mg-, Zn- and Co-based LDH phases. The data point for Mg(OH)_2_ is an average value from six structure determinations: Catti *et al.* (1995 ▶), Černý *et al.* (1995 ▶), Chakoumakos *et al.* (1997 ▶), Desgranges *et al.* (1996 ▶), Isetti (1965 ▶), Zigan & Rothbauer (1967 ▶). The other data points for β-*M*(OH)_2_ phases are from: Kazimirov *et al.* (2010 ▶, Ni(OH)_2_); Pertlik (1999 ▶, Co(OH)_2_); Parise *et al.* (2000 ▶, Fe(OH)_2_); Christensen & Ollivier (1972 ▶, Mn(OH)_2_); Bertrand & Dusausoy (1970 ▶, Cd(OH)_2_); Busing & Levy (1957 ▶, Ca(OH)_2_). There are two data points for β-Zn(OH)_2_: one calculated from Baneyeva & Popova’s (1969 ▶) structure that is labelled ‘Zn cBan’ and a second from Richardson (2013 ▶) that is labelled ‘Zn c1’. Unfilled circles represent the values for the LDH phases and the dotted line is the linear regression fit for those data (the equation is given in the text).
